# Cytotoxic Lesions of the Corpus Callosum (CLOCCs) in a Patient with Epstein–Barr Infection: A Case Report and Literature Review

**DOI:** 10.3390/brainsci15030260

**Published:** 2025-02-28

**Authors:** Ilona Kopyta, Jadwiga Siemek-Mitela, Maria Damps, Magdalena Machnikowska-Sokołowska, Katarzyna Gruszczyńska

**Affiliations:** 1 Department of Paediatric Neurology, School of Medicine, Medical University of Silesia, 40-752 Katowice, Poland; ikopyta@sum.edu.pl; 2 Department of Anesthesiology and Intensive Care, Upper Silesian Child Health Centre, Faculty of Medical Sciences in Katowice, Medical University of Silesia, 40-752 Katowice, Poland; jsiemek-mitela@sum.edu.pl; 3 Department of Diagnostic Imaging, Radiology and Nuclear Medicine, Faculty of Medical Sciences in Katowice, Medical University of Silesia in Katowice, 40-752 Katowice, Poland; magdams@onet.pl (M.M.-S.); kgruszczynska@sum.edu.pl (K.G.)

**Keywords:** corpus callosum, Epstein–Barr virus, magnetic resonance imaging, seizures, splenium

## Abstract

**Background:** Cytotoxic lesions of the corpus callosum (CLOCCs) are a rare disorder of various etiologies referred to as transient lesions of the splenium of the corpus callosum, with a usually mild clinical course. Epstein–Barr virus (EBV) is one of the factors potentially responsible for triggering this abnormality. **Results**: The authors present the case of a 15-year-old girl, so far without any health burden, who suffered from severe CLOCCs with the etiology of EBV. The patient was admitted to hospital because of hepatosplenomegaly and hypertransaminasemia. Her condition rapidly deteriorated—she had seizures with respiratory failure, requiring treatment in the PICU. The first MRI (magnetic resonance imaging) scan showed changes in the hippocampus, and, in the early control, changes like those of CLOCCs; in follow-up studies (one and three months after the onset of respiratory failure), a gradual incomplete regression of the changes in the corpus callosum was seen. Her clinical condition improved quickly, with no seizures during the follow-up and no signs of focal CNS deficits. Cases of CLOCCs are reported as a secondary syndrome connected with many disease entities (e.g., toxic, infectious, and metabolic). The clinical presentation ranges from asymptomatic to severe cases demanding intensive treatment. The diagnosis is determined via an MRI examination. **Conclusions**: The general prognosis for CLOCCs is good, though the normalization of a brain MRI can take several months. As the only method of showing CLOCCs, MRI is the imaging gold standard. Still, clinical abnormalities often precede radiological changes, as was the case with the reported patient.

## 1. Introduction

The corpus callosum (CC) is the largest commissure in the human brain. It consists of circa 200 million fibers forming white matter tracts responsible for communication between the cerebral hemispheres. It permits the interaction, coordination, and specialization of several brain functions. Anatomically, the CC consists of four main parts: the rostrum, genu, body, and splenium. A more detailed division distinguishes seven subareas: the rostrum, genu, rostral body, anterior midbody, posterior midbody, isthmus, and splenium. The CC originates around the eighth week of gestation, and its development is strictly connected with the growth of the corresponding brain parts. Myelination occurs throughout childhood and adolescence [1].

### 1.1. Epidemiology

The first reports of transient lesions with diffusion restrictions were published in the 1990s. They were referred to as mild encephalitis/encephalopathy with reversible splenial lesion (MERS), reversible splenial lesion syndrome (RESLES), transient splenial lesions (TSLs), and finally cytotoxic lesions of the corpus callosum (CC)—(CLOCCs). The incidence of CLOCCs is unknown, but it is considered a rare disorder [2].

### 1.2. Etiology and Pathophysiology

CLOCCs are secondary afflictions of the CC and are predominantly reversible. Etiological factors can be divided into infection-, trauma-, metabolic-, and other (drug/toxin, vascular) associated causes (Table 1), the most common being infection-associated causes both in adult and pediatric patients. This group includes viral and bacterial pathogens, with plasmodial infections being more sporadic. Principally, infection-associated causes predominate in children. In adults, there is an observed second group of entities associated with CLOCCs connected to drugs and toxins [3,4,5,6]. Problems with identifying entities associated with CLOCCs occur in many patients (both in adult and pediatric groups).

The pathophysiology of CC lesions is hypothesized to result from cytokinopathy created by the cascade of the inflammatory process, both in the humoral and cellular mechanisms [7]. It increases the concentration of glutamate in the extracellular fluid; the high number of glutamate receptors in the corpus callosum explains the particular sensitivity of this structure to this disorder and the penetration of water into astrocytes and neurons [8,9]. It leads to cytotoxic edema. Different etiologies probably converge on the release of pro-inflammatory cytokines in the brain, including interleukin-1, interleukin-6, tumor necrosis factor, and glutamine acid [10], resulting in a common molecular pathway with the same tissue injury and radiological findings despite various triggering clinical conditions [11].

### 1.3. Clinical Manifestations, Diagnosis, and Treatment

The clinical symptoms of patients with transient splenial lesions are nonspecific and depend on the underlying disease. Patients with CLOCCs suffer from general and neurological symptoms such as impairment of consciousness, seizures, ataxia, paresthesia, and headaches. Motor deterioration, slurred speech, neck stiffness, coma, tremors, ataxia, somnolence, dysarthria, visual disturbance, and dizziness have also been reported. Nonspecific clinical features include fever and vomiting. Furthermore, CLOCC-like diagnostic findings can be seen in the magnetic resonance imaging (MRI) examinations of patients without any clinical problems [2,12].

Diagnosis is based on the results of neuroimaging—MRI. Pathognomonic signs usually manifest as ovoid, homogeneous, non-hemorrhagic, and well-demarcated lesions detected in the middle part of the splenium (Type I) and/or more extensive and poorly defined lesions, extending throughout the splenium (described as “Boomerang sign”), callosal radiation, or even parietal subcortical WM (Type II) [13]. Extra-splenial callosal lesions are essentially seen in the pediatric group. Extra-callosal involvement is described in some patients; it may predict an irreversible character of the injury. Lesions are usually hyperintense on T2-weighted and FLAIR sequences, are hypointense or isointense on T1-weighted images, present restricted diffusion, and are not visually enhanced after a contrast injection [2,13,14].

Differential diagnostics involve, among others, ischemic lesions, neuroinflammatory diseases such as acute disseminated encephalomyelitis (ADEM) or multiple sclerosis (MS), neoplasms, and rare metabolic encephalopathies [2,15].

### 1.4. Outcomes and Follow-Up

In most patients, there is a complete regression of the changes in the corpus callosum within a month [16]. An MRI follow-up is usually performed up to several months after diagnosis. Patients with persistent lesions constitute a fractional part [2], and published data cite normal neurodevelopment after recovery [6].

The authors present the case of a 15-year-old girl so far without any health burden, who suffered from severe CLOCCs with the etiology of an Epstein–Barr virus infection. To the best of the authors’ knowledge, few cases of CLOCCs with such an etiology have been described in the literature.

## 2. Case Report

The 15-year-old girl was born from an unburdened pregnancy and childbirth, showing the proper course of psychomotor development and achieving good results in labor. The first symptoms of disease were nausea, vomiting, fever, and cough, with the child reporting imbalances and vision disturbances. For this reason, computed tomography of the head with contrast was performed at a regional hospital, revealing that the presentation of the CC was normal. However, laboratory tests revealed hypertransaminasemia and abdominal ultrasound revealed hepatosplenomegaly. For this reason, the child was transferred to the gastroenterology ward of the tertiary hospital. After less than a day’s stay in the gastroenterology department, the child’s condition deteriorated significantly, with rapidly increasing severity disturbances and seizure with respiratory failure (Glasgow score 7), which was an indication for the child’s transfer to the ICU (intensive care unit). An initial brain MRI, which was performed on the first day of neurological symptoms, did not show any changes in the corpus callosum. The most significant deviations in the laboratory tests were hypertransaminasemia, an elevated concentration of C-reactive protein, an increase in D-Dimers, and a prolongation of aPTT (activated partial thromboplastin time) and, in the blood count, leukocytosis with a predominance of lymphocytes and a significant presentation of monocytes. In the cerebrospinal fluid image, slight lymphocytic pleocytosis and an increased protein concentration were observed (the number of cells was 17/uL, protein concentration: 119 mg/dL, glucose: 60 mg/dL, and chloride: 128 mmol/L).

The culture was negative, and antigens typical of meningitis were not detected. A positive monotest and a high titer of antibodies against EBV in the blood serum, together with rashes on the skin of the whole body that appeared on the 3rd day of stay in ICU, organomegaly, and the results of laboratory tests determined the diagnosis of mononucleosis.

**Figure 1:**
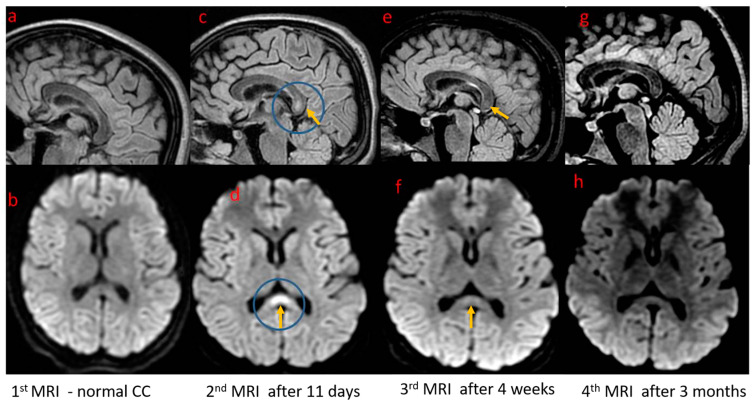
Brain MRI: T2 FLAIR sequence, in the sagittal plane (upper row) and DWI in the axial plane (lower row) showing the appearance and resolution of the abnormal signal in the corpus callosum (CC). (**a**,**b**) Initial MRI performed at the day of the onset of neurological symptoms (which precede the MRI one’s)—normal appearance of the CC. (**c**,**d**) Increased signal in the CC splenium with diffusion restriction 11 days after the onset of symptoms. (**e**,**f**) Partial resolution of changes after 1 month. (**g**,**h**) Further, nearly complete resolution after 3 months. MRI scans were performed using Artist 1.5 T GEM (General Electric, GE Healthcare, Milwaukee, WI, USA) according to the standard protocol comprising the most important sequences for CLOCC diagnosis: T2 Flair and DWI (diffusion-weighted imaging). Arrows and circle—corpus callosum.

In the ICU, the girl was treated with a wide spectrum of antibiotics, acyclovir, osmotic diuretics, and sedation medicines. She was intubated, and mechanical ventilation was initiated. The child’s general condition, in terms of regaining consciousness and achieving full respiratory and circulatory capacity, quickly improved eight days after admission; hence, the child continued treatment in the neurology department. A control MRI was performed 11 days after the onset of the neurological symptoms; it showed an abnormal signal in the corpus callosum meeting the CLOCC criteria type I. A follow-up MRI was performed within a month of the first examination, showing a partial regression of the corpus callosum changes. One month after the onset of symptoms, the child presented no changes in the neurological examination. Laboratory tests were completely normally, as was the ultrasound image of the abdominal organs. In the EEG-9 recording carried out two weeks after the onset of status epilepticus and monitored one month after the onset, there was a symptomatic absence of seizure activity. In the absence of further seizure incidents, no antiepileptic drugs were used; only a thiopental coma was necessary during the stay in the ICU. After 11 days in the neurology department, the girl was discharged from the hospital. Three months later, she was admitted to the hospital again to perform control examinations. The child was in good condition, without any neurological abnormalities. An MRI was performed, which showed further regression but not a complete resolution of the corpus callosum changes (Figure 1).

## 3. Discussion

Epstein–Barr virus (EBV) belongs to the family of Herpesviridae [3]. This virus usually causes infections called mononucleosis, which are usually mild and self-limited [11]. Neurological complications of EBV infection, most typically, concern the situation of the acute infection; however, there are also reports of the reactivation of infection after different lengths of time following acute illness. About 1–5% of patients who develop an acute EBV infection present neurological symptoms, including meningitis and/or encephalitis, acute disseminated encephalomyelitis, cerebellitis, and cranial paralysis [3,17]. A 10-year observational study of EBV encephalitis by Doja et al. showed a wide spectrum of the course and clinical symptoms of the disease [18]. Two of the twenty-one patients included in the follow-up were in a life-threatening condition and died. Caruso et al. described five cases of children with severe permanent neurological defects [19].

The corpus callosum is particularly vulnerable to damage by various (metabolic, infectious, inflammatory, and traumatic) etiologies due to the large number of glutamate receptors [5,20]. Regardless of the causative factor (infection, trauma, and metabolic factors), macrophages and monocytes are activated, and the secretion of interleukins 1 and 6 increases, which in turn causes T cell induction [21]. Globally, it leads to the damage of the blood–brain barrier and an increase in the secretion of tumor necrosis factor by endothelial cells [7]. Astrocytes produce a high quantity of glutamate and are responsible for blocking their reuptake; they are stimulated by interleukin 1, and, as a result, the concentration of glutamate in the extracellular space can increase up to a hundredfold [22]. Furthermore, microglia take part in this process and can start demyelination. This situation is called cytokinopathy, and the high concentration of susceptible receptors in the corpus callosum is the cause of the cytotoxic onset of edema in this structure of the brain [23,24,25].

Laboratory diagnoses in cases of a suspected acute EBV infection usually consist of detecting the presence of the genetic material of the virus by PCR [15]. In the case of our patient, the clinical picture with symptoms of fever, nausea, and vomiting, followed by organomegaly in the ultrasonography examination and the results of laboratory tests (features of liver cell damage and monocytic smear in the peripheral blood image), as well as a positive monotest and the presence of antibodies to EBV in an ELISA test, determined the diagnosis. The rash appeared around a week after the first nonspecific symptoms of the disease appeared during the patient’s stay in the ICU, which complements the clinical characteristics of EBV infection. At the same time, the patient did not present any symptoms of hemophagocytic lymphohistiocytosis, which is one of the possible hematological complications of EBV infection. Hemophagocytic lymphohistiocytosis predisposes to cytokinopathy and CLOCCs because of the high level of cytokines [26,27].

The images of the brain in the magnetic resonance imaging, i.e., initially a normal corpus callosum, followed by changes appearing in the control MRI and their subsequent gradual disappearance in the control tests, are also consistent with the CLOCC picture described in patients with acute neurological complications of EBV infection [28,29,30].

If the EBV etiology of infection is proven, especially a severe course with complications of the central nervous system, the proposed treatment is acyclovir and steroids; however, their effectiveness is variable. In the case of our patient, until the results of the negative bacteriological tests of blood and cerebrospinal fluid, as well as antigens typical of meningitis, were obtained, we used broad-spectral antibiotics with ceftriaxone, vancomycin, and acyclovir. The antibiotic treatment was then discontinued.

The patient’s seizure state resulted in significant disturbances of consciousness, which contributed to the occurrence of acute respiratory failure requiring the use of mechanical ventilation and treatment in the ICU.

The treatment of CLOCCs is symptomatic and focuses on treating the primary disease. In the case of severe CLOCCs, immunomodulatory treatment may be considered. This procedure was implemented with good results in a patient with CLOCCs due to a Mycoplasma pneumoniae infection accompanied by neuroinfection, as described by Lu et al. [27]. A similar procedure was used with good effect in a case with a severe form of COVID-19 infection [31].

After recovery, our patient did not present any deviations in the neurological examination and the image of the abdominal organs in the ultrasound assessment, nor in the results of laboratory tests, which completely normalized, nor did she present any further seizure incidents. A partial but significant regression of the corpus callosum changes was shown in neuroimaging.

Our patient’s initial disease was mononucleosis, i.e., an acute infection caused by the Epstein–Barr virus. Chronic infection can lead to prolonged symptoms due to chronic inflammation, secondarily causing changes in the corpus callosum. Such observations were reported in the study by Voitiuk et al. [32].

A differential diagnosis for CLOCCs and EBV encephalitis is important primarily because of the different treatments and prognosis. Clinical symptoms during CLOCCs tend to be self-limiting, while changes in the magnetic resonance image usually regress with some delay. In our patient, changes in the MRI persisted with a complete remission of the clinical symptoms, consistent with previously published data [6,33]. In turn, EBV encephalitis, despite the predominantly mild clinical course in relation to encephalitis of other etiologies, may be associated with a prolonged manifestation of clinical symptoms and the risk of permanent neurological complications. Cheng H et al., in their study on EBV infections, noted permanent neurological damage in 14% of patients. The following observations were noted in the above-cited study: cases of neuroinfection were not associated with symptoms of mononucleosis, and only 41.5% of patients with CNS involvement had abnormal MRI scans [34]. The authors of another study also observed a lack of symptoms typical of mononucleosis in most patients with EBV encephalitis [35]. Our patient initially had symptoms of mononucleosis and was subsequently diagnosed with CLOCCs (secondary to EBV infection). It is also worth recalling that a characteristic MRI scan is essential for diagnosing CLOCCs. The fundamental difference between the diseases mentioned is the nature of the changes: primary in EBV encephalitis and secondary in CLOCCs. The coexistence of both diseases may make it difficult to arrive at the right diagnosis, i.e., to determine the dominant medical problem in an individual case.

## 4. Conclusions

In conclusion, CLOCC cases are reported as a secondary syndrome connected with many disease entities. They can be connected with infections (e.g., COVID-19), seizures, antiepileptic treatment, metabolic disturbances, thermogenic drugs, malignancies, SAH, and trauma. Clinical presentation can involve asymptomatic cases and severe conditions demanding intensive treatment. It is important to differentiate primary and secondary (CLOCCs) lesions in the corpus callosum. The general prognosis for CLOCCs is good, though the normalization of the brain MRI can take several months. As the only method of showing CLOCCs, MRI has become the imaging gold standard; however, clinical abnormalities often precede radiological changes, as was the case with the reported patient.

## Figures and Tables

**Table 1:** Diseases/syndromes associated with CLOCCs.

Infections	Metabolic Causes	Vascular Causes	Drug/Toxin Causes	Trauma
**Viral:** COVID-19, Influenza A, EBV, RSV, HHV6, rotavirus, adenovirus, mumps, and dengue **Bacterial:** Mycoplasma pneumoniae, Streptococcus pneumoniae, Staphylococcus aureus, Enterococcus faecalis, Escherichia coli, and Legionella **Plasmodial:** Plasmodium falciparum	Hyponatremia Hypo/hyperglycemia Thyroid crisis Acute kidney failure Alcoholism Extrapontine myelinolysis Central pontine myelinolysis Hepatic encephalopathy Hyperammonemia Hypernatremia Malnutrition Wernicke’s encephalopathy Wilson’s disease Marchiafava–Bignami disease	Subarachnoid hemorrhage Stroke Kawasaki disease	Antiepileptic Neuroleptic Carbamazepine Olanzapine Chemotherapy Dietary supplement Metronidazole Immunomodulatory drugs Toxins Corticosteroids Mumps vaccination	Diffuse axonal injury
**Neoplasms**	**Seizures**	**Autoimmune**	**Others**	
Lymphoma Metastases Glioblastoma Insulinoma Melanocytoma	Epilepsy	Anti-GFAP encephalitis Systematic lupus erythematosus Anti-NMDA encephalitis Antivoltage-gated potassium channel antibodies	Alcoholism, COVID-19 vaccine, deep brain stimulation, high-altitude sickness, migraine, parkinsonism, acute encephalopathy in congenital adrenal hyperplasia, acute altitude sickness, eclampsia, hemolytic uremic syndrome, posterior reversible encephalopathy syndrome, and postpartum cerebral angiopathy	

## Data Availability

The data presented in this study are available on request from the corresponding author due to the confidentiality of medical records.
